# Tumor-Suppressive Cross-Linking of Anti-*T. cruzi* Antibodies in Acute Lymphoblastic Leukemia

**DOI:** 10.3390/ijms25158307

**Published:** 2024-07-30

**Authors:** Víctor Alberto Maravelez Acosta, María del Pilar Crisóstomo Vázquez, Leticia Eligio García, Luz Ofelia Franco Sandoval, Denia Castro Pérez, Genaro Patiño López, Oscar Medina Contreras, Enedina Jiménez Cardoso

**Affiliations:** 1 Laboratorio de Investigación en Parasitología, Hospital Infantil de México Federico Gómez (HIMFG), Dr. Márquez 162. Col Doctores, Cuauhtémoc, México City 06720, Mexico; maravelez@hotmail.com (V.A.M.A.); mcrisostomo@himfg.edu.mx (M.d.P.C.V.); leligio@himfg.edu.mx (L.E.G.); lofrancosandoval@gmail.com (L.O.F.S.); cpdeni@hotmail.com (D.C.P.); 2 Unidad de Investigación en Inmunología y Proteomica, Hospital Infantil de México Federico Gómez (HIMFG), Dr. Márquez 162. Col Doctores, Cuauhtémoc, México City 06720, Mexico; gpatino@himfg.edu.mx; 3 Unidad de Investigación Epidemiologica en Endocrinologia y Nutricion, Hospital Infantil de México Federico Gómez (HIMFG), Dr. Márquez 162. Col Doctores, Cuauhtémoc, México City 06720, Mexico; omedina@himfg.edu.mx

**Keywords:** acute lymphoblastic leukemia, *Trypanosoma cruzi*, anti-*T. cruzi* antibodies, tumor-suppressive cross-linking

## Abstract

Parasites have been associated with possible anticancer activity, including *Trypanosoma cruzi*, which has been linked to inhibiting the growth of solid tumors. To better understand this antitumor effect, we investigated the association of anti-*T. cruzi* antibodies with B cells of the acute lymphoblastic leukemia (ALL) SUPB15 cell line. The antibodies were generated in rabbits. IgGs were purified by affinity chromatography. Two procedures (flow cytometry (CF) and Western blot(WB)) were employed to recognize anti-*T. cruzi* antibodies on SUPB15 cells. We also used CF to determine whether the anti-*T. cruzi* antibodies could suppress SUPB15 cells. The anti-*T. cruzi* antibodies recognized 35.5% of the surface antigens of SUPB15. The complement-dependent cytotoxicity (CDC) results demonstrate the cross-suppression of anti-*T. cruzi* antibodies on up to 8.4% of SUPB15 cells. For the WB analysis, a band at 100 kDa with high intensity was sequenced using mass spectrometry, identifying the protein as nucleolin. This protein may play a role in the antitumor effect on *T. cruzi*. The anti-*T. cruzi* antibodies represent promising polyclonal antibodies that have the effect of tumor-suppressive cross-linking on cancer cells, which should be further investigated.

## 1. Introduction

In recent years, Mexico has had one of the highest incidence rates of childhood ALL in Latin America, accounting for 80% of leukemia cases in children and 20% of leukemia cases in adults [1]. Globally, in 2018, leukemia ranked as the 15th most diagnosed cancer, with 437,033 cases and 309,006 mortalities, making it the 11th leading cause of death due to malignant disorders. The geographic distribution of leukemia is worldwide, with higher prevalence and overall mortality in the more developed countries [2]. Of 2403 children with cancer who were treated at the Ministry of Health in Mexico during 2010, 50% suffered from ALL [3]. Mexico observes a higher incidence than the United States. In Mexico City (CDMX), for children under 15 years of age, the age-standardized incidence rate worldwide (ASIRw) was 63.3 (cases per million) for ALL and 53.1 for ALL. By immunophenotype, the ASIRw was 47.3 for B-cell ALL and 3.7 for T-cell ALL [4]. Among Hispanics, the incidence of childhood ALL is 55.0 cases per million children under 15 years of age. These rates are higher than those reported for other ethnic groups, such as White, Asian-Pacific, and African American [5]. The incidence rate of childhood ALL in CDMX has continued to be among the highest in the world, at over 60 cases per million [4]. 

Additionally, research results highlight the heterogeneous distribution of the incidence rates among different municipalities in CDMX. These data suggest the possible roles of environmental, epigenetic, or lifestyle factors in ALL development among Mexican children. It is important to conduct further research studies to examine the relationships between these factors and ALL risk in different areas [4]. The available treatments have increased the cure rate from 10% to 80–90% in industrialized countries in the past 50 years [6]. This is in unfortunate contrast to the rates in the Mexican child population. In Mexico, as in other developing countries, mortality rates continue to be high, probably because of difficulties in diagnosis and delays in starting treatment [7]. Although the mortality rates of cancer in the pediatric population are low compared to other age groups, it should be considered a priority because, within the age group, it occupies first place. Although ALL has been considered one of the neoplastic diseases that respond well to timely treatment, mortality from this cause indicates access to healthcare and its effectiveness [8]. ALL behaves more aggressively in patients over 40; survival rates in this age group are approximately 30–40%. This is attributed to the greater number of genetic abnormalities in people in this age group [9]. The five-year survival rates exceed 80% for 45,000 children with cancer in high-income countries but are less than 30% for 384,000 children in lower-middle-income countries. The expanding portfolios of new drugs that target the biological mechanisms driving the growth of pediatric cancers are also starting to contribute to improved cure rates in high-income countries. This is not the same for the lower-middle-income countries, and there is a survival difference of up to 45% for ALL between the high-income and lower-middle-income countries [10]. This makes it challenging to treat and cure people in time. 

**Figure 1:**
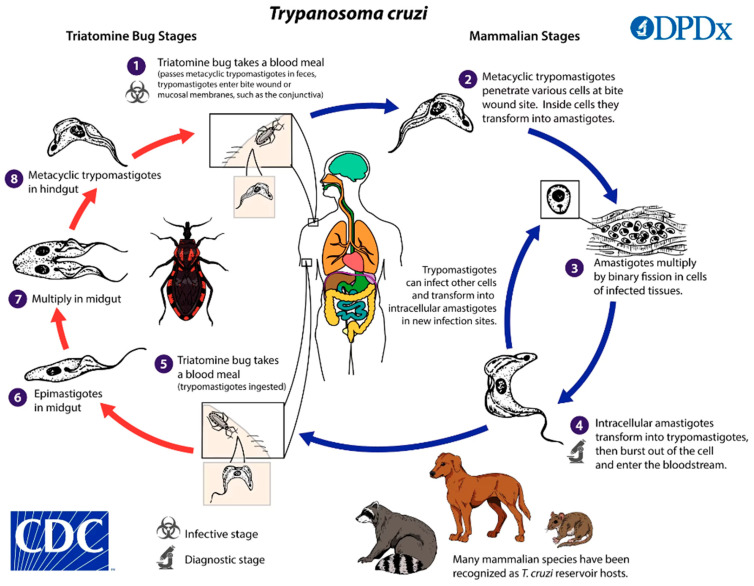
Life cycle of *Trypanosoma cruzi*.

*T. cruzi* is the etiological agent of American trypanosomiasis, or Chagas disease. It is an endemic disease of Latin America and, therefore, is present in Mexico. It is a multisystemic disorder that affects the cardiovascular, digestive, and central nervous systems [11]. Chagas disease is one of the 20 most “neglected tropical diseases”, as defined by the World Health Organization (WHO) [12]. About 6–7 million people are infected worldwide, with almost 100 million at risk, indicating that this disease is a serious public health issue [13]. In endemic countries like Mexico, Chagas disease is transmitted by blood-sucking insects such as triatomines (kissing bugs). While the insect feeds on the blood of a mammalian host, near the wound, it releases feces that contains metacyclic trypomastigotes, which can enter the host through wounds in the skin or mucous membranes. Within the host, trypomastigotes invade cells near the site of the infection. In the blood, the metacyclic trypomastigotes are contained within a parasitophorous vacuole, which they soon overcome, transforming into amastigotes and replicating intracellularly. Subsequently, the amastigotes will transform into trypomastigotes and will be released into the bloodstream. In this way, they will be increasingly moved through the bloodstream and can transfer to tissues, thus infecting more cells or simply being ingested by the vector. Following ingestion, *T. cruzi* trypomastigotes transform within the midgut of the triatome vector, differentiating into epimastigotes. These epimastigotes multiply and subsequently differentiate into infectious metacyclic trypomastigotes within the hindgut of the vector. While the primary transmission route involves the vector’s feces, other less common transmission mechanisms have been documented. These include blood transfusions, organ transplantation, transplacental transmission from mother to fetus, and, potentially, dietary transmission through food or drinks contaminated with the vector or its feces [14,15] (Figure 1). The life cycle of *T. cruzi* involves three main developmental stages: epimastigote, amastigote, and trypomastigote [16]. While both epimastigotes and amastigotes are capable of division, trypomastigotes are nondividing and represent the parasite’s infective stage. Epimastigotes, residing in the intestines of triatomine insects, are noninfectious to humans. However, they constitute the replicative stage of the parasite, both within the insect vector and in laboratory cultures. This noninfectious nature makes epimastigotes a suitable and safe model for in vitro studies, facilitating the investigation of *T. cruzi* biology. Despite their noninfective status, epimastigotes possess significant antigenic properties. Studies employing total epimastigote protein extracts have demonstrated their role in eliciting an antitumor effect against *T. cruzi* [17,18,19], highlighting their immunogenic potential. Furthermore, epimastigote proteins are commonly utilized in commercially available ELISA kits for Chagas disease diagnosis [20], indicating shared antigenic determinants between epimastigotes and trypomastigotes. The CL Brener (CLB) strain of epimastigotes, a widely studied model, is a valuable tool for exploring these antigenic properties. This strain, alongside others, offers a unique opportunity to unravel the intricate interplay between epimastigote antigens and the host immune response, contributing to the development of novel diagnostic and therapeutic strategies for Chagas disease.

The potential interplay between parasites and cancer has been a subject of scientific interest since the 1930s [21]. While some parasites have been implicated in tumorigenesis, others have demonstrated the capacity to inhibit cancer cell growth [22]. Notably, *T. cruzi* has garnered attention for its potential oncoprotective effects in animal models and in vitro experiments [17,18,19,21,22,23,24,25,26,27]. *T. cruzi* antigens (including total proteins), anti-*T. cruzi* antibodies, and even live parasites have been shown to exert antitumor effects on neoplastic cells [17,18,19,21,22,23,24,25,26,27]. Specifically, *T. cruzi* infection has been shown to reduce tumor growth in malignant melanoma in a mouse model [23]. Chronic infections exhibited more favorable outcomes than acute infections, suggesting a correlation between the parasite’s invasive capacity and the elicited immune response in mediating oncoprotection [23]. Further evidence supporting the antitumor potential of *T. cruzi* stems from studies demonstrating that antibodies directed against *T. cruzi* inhibit the growth of cancer cells. For instance, antibodies targeting a peptide with homology to human and *T. cruzi* mucins have been shown to induce antibody-mediated cytotoxicity in tumor cells, reducing their viability by up to 50% [24]. Additionally, polyclonal anti-*T. cruzi* epimastigote phase antibodies have been shown to cross-react with colon and breast cancer cells, triggering an immune response involving both humoral and cell-mediated immunity components [17]. Previous studies have implicated *T. cruzi* antigens as potential mediators of an oncoprotective effect, with particular interest in the role of calreticulin. Calreticulin, a chaperone protein residing in the endoplasmic reticulum of eukaryotic cells, has been proposed to exhibit antiangiogenic and tumor growth regression properties [25,26]. *T. cruzi* calreticulin (TcCRT) is known to interact with the complement component C1 after translocation from the endoplasmic reticulum. This interaction is disrupted by cC1qR, a membrane-bound calreticulin found in mammals, inhibiting the complement system [28]. The complement system is crucial for both innate and adaptive immunity, and its inhibition may enhance parasite infectivity. Intriguingly, in vivo studies have demonstrated that subcutaneous peritumoral inoculation of recombinant *T. cruzi* calreticulin (rTcCalr) promotes local T cell infiltration and delays tumor development [29]. The mechanism underlying this effect appears to involve the binding of rTcCalr to TA3 cells, which subsequently facilitates the binding of C1q to the tumor cells through rTcCalr. This interaction enhances phagocytosis of TA3 cells by murine macrophages, suggesting an enhanced antitumor immune response. Furthermore, treatment with rTcCalr reduced the membrane expression of MHC class II, m-Dectin-1, Galectin-9, and PD-L1 on TA3 cells. These molecules are involved in immune regulation and tumor evasion. The modulation of these surface markers by rTcCalr may contribute to increased tumor immunogenicity, further supporting the potential of rTcCalr as a therapeutic agent in cancer treatment. 

While the potential antitumor effects of *T. cruzi* infection have been explored, a definitive link between Chagas disease and cancer protection remains elusive. Existing studies primarily focus on animal models and in vitro analyses, leaving unclear the precise mechanisms underlying any potential antineoplastic activity. Furthermore, the potential for anti-*T. cruzi* antibodies to exert a direct effect on hematological malignancies, such as ALL, a significant public health burden in Mexico, has not been investigated. This study addresses the following crucial questions: (a) Do polyclonal anti-*T. cruzi* antibodies bind to B cell antigens associated with ALL? (b) Do anti-*T. cruzi* antibodies contribute to the cytotoxicity of ALL cells? (c) Can we identify specific protein targets responsible for antibody recognition and cytotoxicity? Previous studies investigating the tumor effects of *T. cruzi* have highlighted the parasite’s capacity to induce a complex antitumor response involving both cellular and humoral components of the immune system [17]. This finding suggests a potential for beneficial interplay between parasite infection and tumor growth. However, molecular targets for antileukemic activity based on this potential association remain unexplored. Addressing these questions will provide novel insights into the complex interplay between Chagas disease, the immune response, and hematological malignancies, potentially paving the way for novel therapeutic strategies targeting ALL.

## 2. Results

The epimastigotes *T. cruzi* CLB isolate was grown in LIT medium and, after 48 h, it was harvested and washed. Antigens were obtained, and antibodies were induced in New Zealand rabbits under an immunization scheme. The IgG was purified from the hyperimmune sera. The final concentration of la IgG (anti-*T. cruzi* antibodies) was 8.8 µg/µL.

### 2.1. Recognition of Anti-T. cruzi Antibodies on the Membrane of SUPB15

To investigate the recognition of *T. cruzi*-specific antibodies by the membrane protein SUPB15, FC analysis was performed. We evaluated nonpermeabilized SUPB15. Anti-*T. cruzi* antibodies (8.8 μg/µL) were diluted (1:300) and incubated with SUPB15-expressing cells. The binding of these antibodies was detected using Alexa Fluor 488-conjugated anti-rabbit IgG antibodies (1:300). A total of 100,000 cells were analyzed for each condition using a BD FACSCanto II flow cytometer (BD Biosciences, San Jose, CA, USA). Data analysis was performed with FlowJo v10.0.7 software (Tree Star, Ashland, OR, USA).

**Figure 2:**
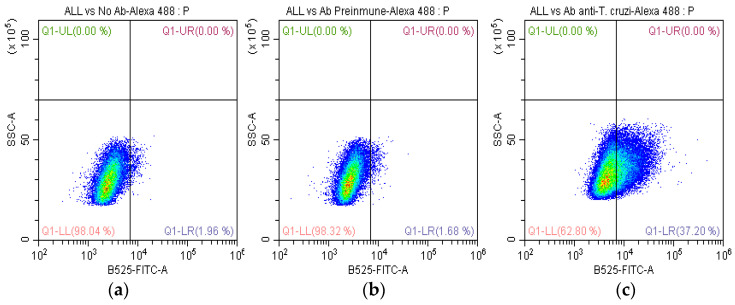
Recognition of membrane proteins of SUPB15 with anti-*T. cruzi* antibodies by FC. The quadrant Q1-LR shows the percentage of cells that recognize anti-*T. cruzi* antibodies. (**a**) SUPB15 and Alexa 488 (with no Ab), (**b**) SUPB15 with preimmunized antibody and Alexa 488, and (**c**) SUPB15 with antibodies anti-*T. cruzi* and Alexa 488 antibodies.

The results indicated that 35.5% of SUPB15-expressing cells displayed positive staining for the anti-*T. cruzi* antibodies (Figure 2c). This percentage was significantly higher than the control group, which consisted of SUPB15 incubated with preimmunized IgG (Figure 2b). These findings suggest that a substantial proportion of SUPB15 molecules on the cell surface are recognized by *T.-cruzi*-specific antibodies, highlighting a potential role for this protein in the host immune response to *T. cruzi* infection.

### 2.2. Evaluation of Anti-T. cruzi Antibodies with CDC

**Figure 3:**
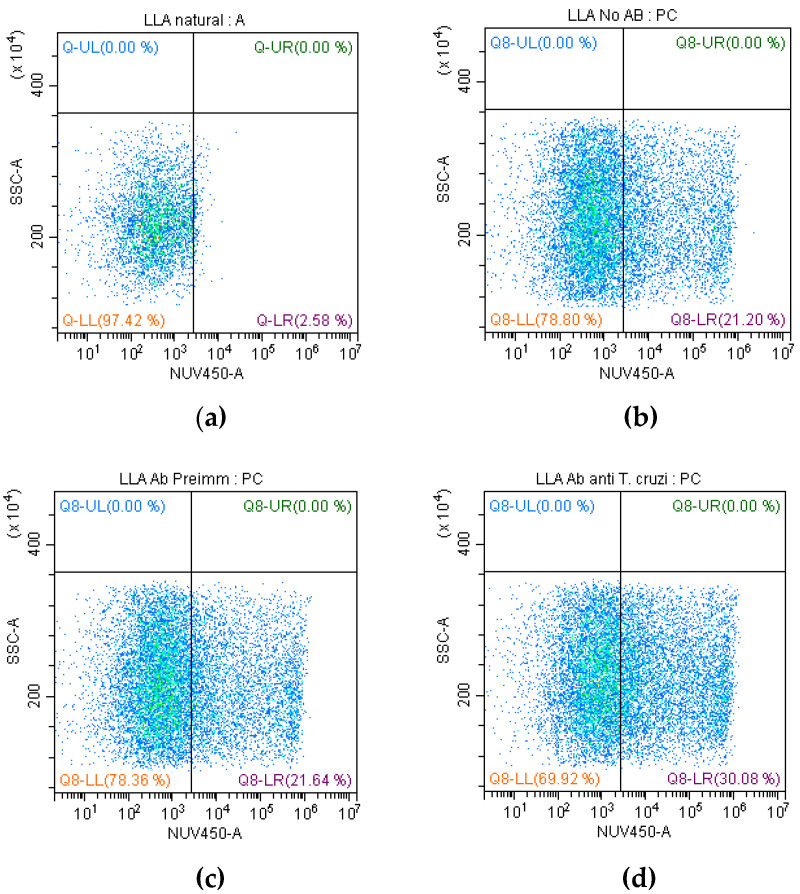
Determination of the CDC with anti-*T. cruzi* antibodies; the quadrant Q8-LR shows the percentage of cytotoxic. (**a**) SUPB15 autofluorescence, (**b**) SUPB15 and complement (with-out Ab), (**c**) SUPB15 with preimmunized antibody and complement, and (**d**) SUPB15 with antibodies anti-*T. cruzi* and complement. Orange CMRA and Blue live/dead staining.

The CDC was evaluated by flow cytometry. We examined the impact of CDC of anti-*T. cruzi* antibodies on SUPB15 (Figure 3). We used proliferation (Orange CMRA-labelled SUPB15 cells) and viability (Blue LIVE/DEAD) (Invitrogen, Eugene, OR, USA) stainings to confirm cytotoxicity. Anti-*T. cruzi* antibodies (8.8 μg/µL) were diluted (1:300). The levels of cytotoxicity in the SUPB15 were higher in the presence of *T. cruzi* antibodies and complement (Figure 3d) than in controls with preimmunized IgG (Figure 3c) and without antibodies (Figure 3b). Our results demonstrate that anti-*T. cruzi* antibodies suppress the SUPB15 up to 8.4%. Cells were acquired using a (Becton Coulter, New York, NY, USA) flow cytometer, and the data were analyzed using FlowJo v10.0.7 (Tree star, Ashland, OR, USA) software.

### 2.3. Recognition of Anti-T. cruzi Antibodies on the Total Proteins of SUPB15

**Figure 4:**
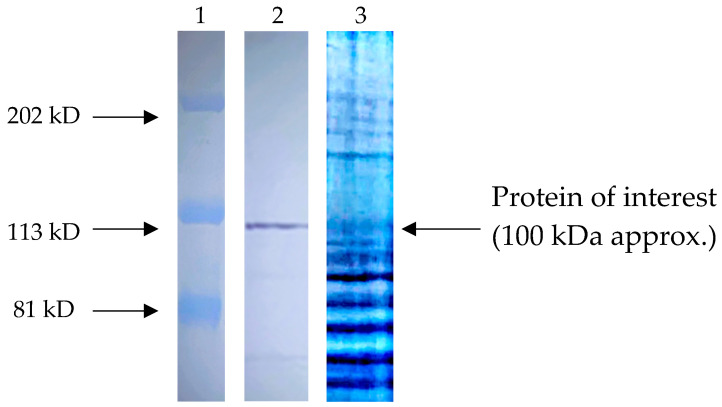
Protein profile and recognition of SUPB15 with anti-*T. cruzi* antibodies by WB. Shown: (**1**) Molecular weight. (**2**) Western blot, protein extract of SUPB15 (1000 μg); preparation was separated by SDS_PAGE 12% and electro-transferred to nitrocellulose membranes, reactivity was carried out with the anti-*T. cruzi* antibodies 1:100, and anti-rabbit IgG coupled to peroxidase 1:10,000 was used for immunostaining and rebelled with 4-chloro-naphthol. (**3**) Electrophoretic separation: protein extract of SUP15 (1000 μg) separated by SDS-PAGE 12% for 2 h, Coomassie stains.

To identify SUPB15 proteins recognized by anti-*T. cruzi* antibodies, we employed a combination of Western blotting and mass spectrometry. We performed Western blot analysis using total protein extracts of SUPB15 against a panel of anti-*T. cruzi* antibodies. A prominent band corresponding to a 100 kDa protein fragment was consistently detected (Figure 4). This finding suggests that anti-*T. cruzi* antibodies may effectively target specific antigens present within SUPB15.

### 2.4. Identification of the Most Abundant Proteins of a 100 KDa Fragment

**Figure A1:**
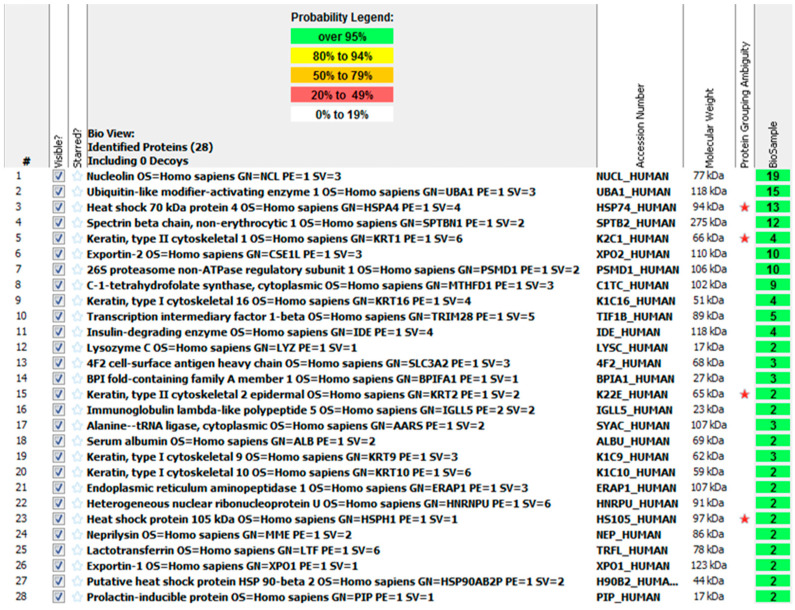
SUPB15 proteins of the antigenic site recognized by anti *T. cruzi* antibodies. The 28 proteins identified from the protein peptides present a 100 kDa antigenic site by mass spectrometry, are shown by red stars.

The 100 kDa protein band, visualized in the SDS-PAGE (Figure 4), was excised and submitted to the University Laboratory of Proteomics, IBT-UNAM (Morelos, Mexico), for further analysis. Mass spectrometry analysis was performed using a quadrupole-TOF Impact II mass spectrometer. The acquired mass spectra were processed using Data Analysis software 5.0 (Bruker Daltonics, Billerica, MA, USA) and subsequently searched against the Swiss-Prot protein database employing the Mascot search engine. This analysis identified 28 proteins (Figure A1 in Appendix A). Among these, nucleolin exhibited the highest number of identified peptides (19) within the characterized antigenic site. Notably, nucleolin has been previously reported in the SUPB15 cell line. 

### 2.5. Identification of the Nucleolin in SUPB15

**Figure 5:**
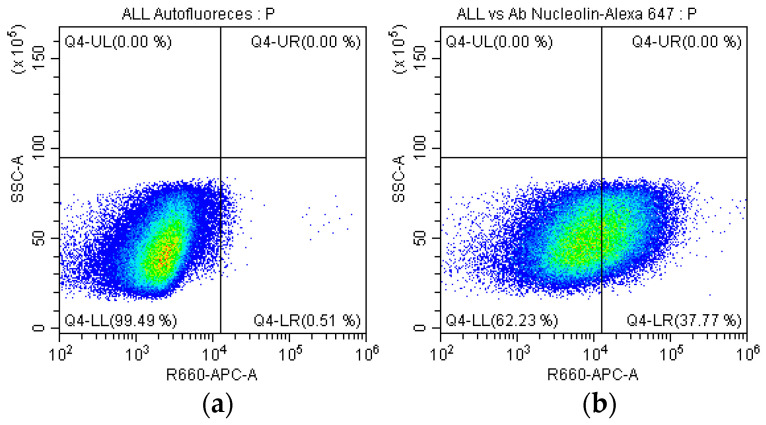
Determination of nucleolin in SUPB15; the quadrant Q4-LR shows the recognition percentage. (**a**) SUPB15 and Alexa 647 antibodies (autofluorescence); (**b**) SUPB15 with anti-nucleolin and Alexa 647 antibodies.

We confirmed the presence of nucleolin in the plasma membrane of SUPB15. Immunostaining with a mouse monoclonal antibody specific for nucleolin (clone: NCL/902, NeoBiotechnologies, cat. 4691-MSM1-P1) followed by Alexa Fluor 647-conjugated secondary antibody revealed the presence of nucleolin on the plasma membrane of 37.7% of SUPB15 (Figure 5b). CF was analyzed using a BD FACSCanto II flow cytometer (BD Biosciences, San Jose, CA, USA) and FlowJo v10.0.7 software (Tree Star, Ashland, OR, USA). These findings provide evidence for nucleolin’s localization to SUPB15 plasma membrane. 

### 2.6. Evaluation of Anti-Nucleolin Antibodies with CDC

**Figure 6:**
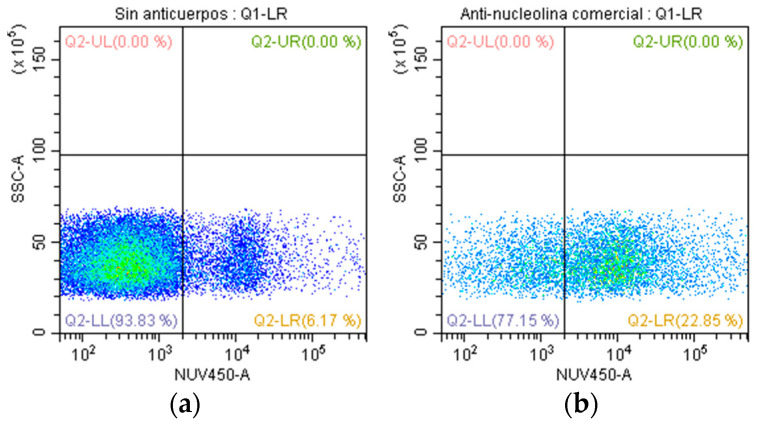
Determination of the CDC by anti-nucleolin monoclonal antibody. The quadrant Q2-LR shows the percentage of cytotoxic cells. (**a**) SUPB15 and complement (with no Ab) and (**b)** SUPB15 with antibodies against nucleolin (clone: NCL/902, NeoBiotechnologies) and complement. Orange CMRA and Blue live/dead staining.

To investigate the cytotoxic potential of anti-nucleolin antibodies, we employed a mouse monoclonal antibody (clone: NCL/902, NeoBiotechnologies, cat. 4691-MSM1-P1) and assessed its impact on the SUPB15 (Figure 6). Our findings revealed a significant level of CDC in SUPB15, with a 16.7% reduction in cell viability observed in the presence of anti-nucleolin antibodies (Figure 6b). This suggests that anti-nucleolin antibodies, through their binding to nucleolin expressed on the surface of SUPB15 cells, effectively engage immune effector cells to induce cell death. These results demonstrate the potential of anti-nucleolin antibodies as a therapeutic strategy for targeting SUPB15, highlighting the crucial role of nucleolin as a potential target for antibody-mediated therapy.

## 3. Discussion

The beneficial association between infection with different parasites and human cancer has been demonstrated in several studies [22]. Similarly, the potential antitumor effect of *T. cruzi* against solid and hematological neoplasms has also been demonstrated [17,18,19,21,22,23,24,25,26,27]. *T. cruzi* has genetic, protein, and antigenic variability, attributed to its antitumor properties [18]. Here, we report a beneficial association between *T. cruzi* antibodies and hematological cancer. Previously, we found that anti-*T. cruzi* antibodies recognize the SUPB15 plasma membrane [27]. First, we investigated whether anti-*T. cruzi* antibodies could recognize lymphoblasts on the membrane of SUPB15 using flow cytometry. We identified 35.5% positive cells (Figure 2). Similar results have been obtained when using anti-*T. cruzi* polyclonal antibodies in colon cancer cells, which were found to have activity in the cytoplasm and cell membrane [17]. Using antibodies directed against synthetic glycopeptides resembling *T. cruzi* and human mucins, 20.5% and 24% recognition rates were achieved in breast cancer cells (MCF-7) [17]. Our results using anti-*T. cruzi* antibodies show higher recognition, possibly because their polyclonal nature has greater coverage. Then, we performed CDC to determine if anti-*T. cruzi* antibodies can cause lymphoblast cell death. We observed 8.4% cytotoxicity compared to the control, using preimmunized IgG (Figure 3). Furthermore, we observed a tumor-suppressive cross-linking of anti-*T. cruzi* antibodies on SUPB15, with a proportion of these anti-*T. cruzi* antibodies reacting against SUPB15 antigens. *T. cruzi* infection can induce a humoral autoimmune response because some parasite proteins mimic human proteins. Molecular mimicry is a mechanism involved in Chagas disease [30]. As a result, anti-*T. cruzi* antibodies may recognize human proteins. This may help identify SUPB15 tumor antigens. Both calreticulin and mucins are *T. cruzi* proteins involved in the antitumor effect [19,25,26]. 

SDS-PAGE analysis of SUPB15 total protein extracts revealed a prominent band at approximately 100 kDa (Figure 4). Subsequent mass spectrometry analysis identified 28 distinct proteins within this band (Appendix A). This relatively high number of identified proteins is likely due to a one-dimensional gel electrophoresis. Among the identified proteins, nucleolin emerged as a particularly notable candidate. This designation is based on two key factors, the high number of peptides identified for this protein and its molecular weight, which aligns closely with the sequenced fragment.

Nucleolin, an isoform of nuclear nucleolin called nucleolin CRA_b, has been found on the surface of PreB-ALL lymphoblasts as a 9-0-acetyl sialoglycoprotein and has been proposed as a marker of Pre-B ALL. The nucleolin found in the Pre-B cell membrane of ALL is in the form of a 9-O-acetylated sialoglycoprotein that can be identified by specific antibodies [31]. Our specific antibodies can identify nucleolin on 37.7% of SUPB15 (Figure 5); this value is acceptable because nucleolin is not always present on the surface of ALL cells [31]. The presence of nucleolin in *T. cruzi* has not been determined. The absence of nucleolin in *T. cruzi* suggests a potential role for the nucleolin identified in SUPB15 in the anti-*T. cruzi* antibody response. This observation may be attributed to a cross-reaction between the mucins of *T. cruzi* and nucleolin in SUPB15. Both proteins share similarities in their membrane localization and ability to incorporate sialic acid into their structure, potentially facilitating this cross-reactivity. The *T. cruzi* glycocalyx, responsible for its surface properties, is predominantly composed of glycoinositolphospholipids (GIPLs) [32] and glycosylphosphatidylinositol (GPI)-anchored mucins [33]. The presence of GPI anchors contributes to the dense packing of mucins, further highlighting their crucial role in *T. cruzi* surface interactions. Carbohydrates constitute approximately 60% of the total mass of mucins [34], emphasizing their significant contribution to the overall structure and function of the glycocalyx. This structural similarity between mucins and nucleolin, particularly in their carbohydrate moieties and membrane localization, could explain the observed cross-reactivity and the potential role of SUPB15 nucleolin in the anti-*T. cruzi* antibody response. Further investigation is required to elucidate this interaction’s exact nature and mechanism.

Epimastigotes and trypomastigotes have approximately the same number of mucin molecules per cell [34]. Other less abundant but distinctive glycoproteins have been described in *T. cruzi*, among them a trans-sialidase [35]. The structure of the O-linked chains in the mucins defines their role in antigenicity and pathogenesis. They perform a crucial function as acceptors of sialic acid from host glycoconjugates in a reaction catalyzed by the parasite’s unique trans-sialidase, an enzyme extensively studied [36]. Two pivotal players in *T. cruzi*, mucins and the trans-sialidase, have been extensively investigated. They were called mucins because of the high sugar content in their O-linked chains, although their structures differ significantly from the human mucins. One of the differences is that GlcNAc, instead of GalNAc, links the O-chain to the protein [37]. Galactose, in the form of β-D-galactofuranose (β-Gal f), is exclusively present in epimastigotes mucins, rendering them highly antigenic. Mucin carbohydrates fulfill crucial functions, the most important of which is the acceptance of sialic acid from the host, a process catalyzed by trans-sialidase, a unique molecule of *T. cruzi.* [38]. Sialylated ligands are strong candidates for interfering with host immune responses, both innate and adaptive [s]. Sialylated mucins mask the antigenic determinants of the parasite, thus protecting the parasite from host attack [34]. Recent studies have shown that, in addition to masking parasite antigens, sialic acid on the surface of *T. cruzi* is also responsible for direct interaction with the host cell’s inhibitory receptor, Siglec-E. This sialylation influences the effectiveness of *T. cruzi* mucins (Tc Muc) inhibitory properties on dendritic cell function through interaction with sialic-acid-binding Ig-like lectins that are predominantly expressed in immune system cells [39]. The interaction mediated by glycoconjugates expressed in parasites and sialic-acid-binding Ig-like lectin-E expressed in host cells may elucidate effects at the interface between parasites and host cells [40]. The sialyl motif of Tc Muc can interact with Ig-like lectins (Siglecs) that bind sialic acid on CD4+ T cells. [40]. In light of this finding, we propose that the SUPB15 surface nucleolin has a receptor involved in the recognition by the structure of some *T. cruzi* mucin. Nucleolin, a protein expressed on the surface of SUPB15 with sialic acid receptors, is another candidate for polyclonal anti-*T. cruzi* antibodies interacting with SUPB15 due to the sialic acid receptors of *T. cruzi* mucins. However, this hypothesis needs to be proven, and, therefore, a mechanism of action cannot be determined yet. We suggest obtaining an immunoconjugate that contains the organic part that incorporates sialic acid and investigating the antigen–antibody interaction in *T. cruzi* and SUPB15. Identifying homologous proteins in the plasma membrane of SUPB15 is crucial for understanding the mechanism of action of anti-*T. cruzi* antibodies in a potential antitumor setting. While cross-reactivity studies can provide initial insights, the limited number of identified targets and the need for further mechanistic investigation necessitates exploring alternative approaches. We propose an alternative strategy to identify potential target proteins by focusing on the homologous proteins present in the plasma membrane of SUPB15. This approach, based on comparative genomics and proteomics, aims to uncover potential targets of anti-*T. cruzi* antibodies that may contribute to their antitumor effect. By analyzing the role of these antibodies in the antitumor effect, we aim to provide evidence supporting the use of anti-*T. cruzi* antibodies as a potential alternative treatment strategy for specific cancers. This research will contribute to developing novel therapeutic approaches for neoplastic diseases. 

In Figure 3, we observed 8.4% cytotoxicity compared to the control; with only two values, it is difficult to establish a robust statistical conclusion, which suggests the need to include more data, and this could be significant since we used polyclonal antibodies, so it is not just one antibody but they contain a wide variety of antibodies. We used a monoclonal antibody specific for nucleolin; with this antibody, the cytotoxicity was 16.7% (Figure 6), although higher than the value obtained with the polyclonal antibodies. However, it cannot be high enough to denote an important value; designing specific antibodies against nucleolin that were more antigenic and cytotoxic would be necessary. Nucleolin is a protein that, although overexpressed in Pre-B ALL cells, is not always present simultaneously in all cells [31]. This makes the binding of antibodies difficult and consequently affects cytotoxicity. The nucleolin of the cell membrane of cancer cells is linked to proliferation, anti-apoptosis, and migration [41,42]. Therefore, nucleolin interacts so that the cancer cell evades the immune system. Cell membrane nucleolin interacts with Fas receptor to prevent Fas-induced apoptosis activated by Fas ligand (FAS-L) in B-cell lymphoma cells. For these reasons, the SUPB15 cytotoxicity possibly induced by anti-*T. cruzi* antibodies is not as significant. Blocking cell surface nucleolin with specific antibodies increases apoptosis, decreases endothelial cell migration, and prevents angiogenesis [43,44]. Nucleolin participates in many functions in cancer cells, making it an important molecular target to stop tumor growth. 

We found recognition of SUPB15 cells by anti-*T. cruzi* antibodies; we postulate that SUPB15 cell surface nucleolin is the interacting protein. Additionally, we propose that this union induces cytotoxicity determined by complement on SUPB15. This raises the following questions: (i) Is there possible a relationship between Chagas disease and the high incidence of B-ALL in Mexico? With Mexico being an endemic country of Chagas disease, (ii) Do anti-*T. cruzi* antibodies represent an antitumor effect or a protumor effect? This can be analyzed in several different ways: (i) all in vitro or in vivo studies in animal models highlight an antitumor effect, such as a reduction in tumor cells where *T. cruzi* intervenes, either through passive or active immunity or due to direct infection of the parasite [17,18,19,21,22,23,24,25,26,27]; it has also been shown that anti-*T. cruzi* antibodies trigger a humoral and cellular response against colon cancer [17]. Studies in humans do not exist, which makes it difficult to answer these questions. (ii) In Mexico, a recent estimate placed the seroprevalence of *T. cruzi* at 3.38% nationally, representing 4.06 million infected people in the country [45]. In total, 88% of the population of Mexico is exposed to *T. cruzi* infection through the vector, given that 19 species of triatomines (of 31 described for the country) commonly invade human homes and 29 have been reported with infection with *T. cruzi* [46,47]. CDMX, one of the most populated cities in the world, has no direct contact with the vector and has one of the highest incidences of B-ALL in children under 15 years of age in the world. Does Chagas disease reduce or increase the risk of B-ALL? This would explain it, at least for CDMX. However, another state located 55 km away, the State of Morelos, is one with the highest incidence of Chagas disease. Unfortunately, there are not enough epidemiological data about B-ALL incidence. In Mexico, estimates of Chagas disease vary widely due to demographic and regional differences [45,48,49]. We propose to study these epidemiological data to explore the question of having anti-*T. cruzi* antibodies and cancer. (iii) Our in vitro studies found that anti-*T. cruzi* antibodies provide minor protection against SUPB15, as determined by CDC. Tumor cells are often resistant to CDC due to mechanisms involving membrane-bound complement regulatory proteins (mCRP). Fc–C1q interaction initiates complement-dependent cytotoxicity (CDC) and generates membrane attack complexes through the assembly of various serum complement molecules, leading to tumor cell lysis [50]. CDC influences the effectiveness of several anticancer antibodies, including rituximab (Rituxan^®^; anti-CD20), the first anticancer antibody approved by the US FDA [51]. Tumor cells express membrane-bound complement regulatory proteins (mCRP; CD35, CD46, CD55, and CD59) that normal cells use to prevent excessive activation of complement cascades in the early stages of an immune response [52,53]. Tumor cells can evade the target cell clearance mechanism mediated by complement of IgG antibodies for survival [54]. mCRP, CD55 accelerates C3 convertase degradation and is overexpressed on the cell surface of several cancers such as leukemia [55]. This evasion mechanism due to mCRP might explain the low CDC value in our study. Rituximab (CD20) was found to cause resistance of cancer cells due to CD55, so one strategy to block CD55 and improve the efficiency of CDC was with an asymmetric bispecific antibody that binds to CD55 and CD20 [56]. This could be implemented in a future study with anti-*T. cruzi* antibodies and consider also blocking other membrane-bound complement regulatory proteins to increase their CDC efficiency. ADCC activity in this study was not evaluated but this could be conducted in future research. VHH single-chain anti-nucleolin antibodies fused to an Fc region of human IgG1 against melanoma cells showed greater ADCC activity than conventional VHH anti-nucleolin antibodies between 2 and 1.7 times. VHH antibodies have the characteristic that they lack light chains and consist of two heavy chains linked to variable domains (VHH), giving them the ability to bind to epitopes that are normally not accessible to conventional antibodies because they are smaller and have greater solubility [57]. Considering these, anti-*T. cruzi* antibodies may represent an antitumor effect that could be seen as more relevant if strategies are incorporated in future studies to make them the most efficient concerning CDC and ADCC. (iv) Nucleolin, a multifunctional phosphoprotein that acts through interaction with various proteins and nucleic acids, plays a crucial role in the production of cytokines in dendritic cells (DC). Nucleic-acid-based adjuvants efficiently induce Th1-type immune responses and CD8 + T cell responses [58,59], for example, CpG ODN (short single-stranded synthetic DNA fragments containing immunostimulatory CpG unmethylated cytosine–phosphate–guanine) and the poly(I:C) (the other type of nucleic-acid-based adjuvant is polyinosinic-polycytidylic acid (poly[I:C])). Poly(I:C) is a synthetic analog of double-stranded RNA [60,61]. CpG ODN and poly(I:C) inhibit the immunostimulatory activity of bacterial DNA and tumor cells. In human DCs, nucleolin also contributed to the binding and internalization of CpG ODN and subsequent cytokine production [62]. Is it likely that anti-*T. cruzi* antibodies can inhibit the immunostimulatory activity of dendritic cells by recognizing nucleolin on the cell surface of dendritic cells? There is no literature on this topic. CpG ODNs and poly(I:C) significantly inhibited the binding of MS-3 (anti-nucleolin antibodies) to the dendritic cell surface. This means there is possibly competition for binding to similar epitopes on nucleolin in dendritic cells [62]. An inhibition of immunostimulatory activity would not be affected at all. This could happen in a similar way with anti-*T. cruzi* antibodies. But it is something that must be verified in future studies. In summary, anti-*T. cruzi* antibodies are recognized by SUPB15 and present tumor-suppressive cross-linking of anti-*T. cruzi* antibodies. It was determined through cross-linking of anti-*T. cruzi* antibodies that we were able to identify nucleolin as a protein that is involved in the interaction. Defining the antitumor effect of anti-*T. cruzi* more broadly in neoplastic cells is a challenge. If more studies continue to be carried out on this effect, the anti-*T. cruzi* antibodies may be used as an alternative in cancer care. Further studies are necessary to explore this possibility.

## 4. Materials and Methods

### 4.1. Parasite Culture and Obtaining the Antigen

Epimastigotes *T. cruzi* CLB isolates were cultured at 28 °C and harvested in exponential growth in LIT medium supplemented with 10% fetal calf serum (Gibco, New York, NY, USA) [63]. Epimastigotes were counted in a Neubauer chamber and viability was assessed with trypan blue. To extract the antigen, subsequently, the cell pellet was resuspended in cold protein lysis buffer (1% Nonidet P-40, 150 mM NaCl, 10 mM Tris-HCl pH 7.6, 10 mM PMSF, 2 mM EDTA, Protinin A 1 µg/mL, Pestatin A 1 µg/mL y Leupeptin A 1 µg/mL) [64] and lysed by freezing for 30 min. The total Ag concentration obtained was determined on a UV-Visible spectrophotometer at 280 nm Epoch (Biotec Instruments, Winooski, VT, USA).

### 4.2. Cell Line of SUPB15 and Obtaining the Antigen

The SUPB15 cell line was obtained from ATCC (Manassas, VA, USA). SUP-B15 is a B lymphoblast cell line isolated from the marrow of a White, 8-year-old, male patient with acute lymphoblastic leukemia. The cell line was cultured in RPMI 1640 media (Gibco, New York, NY, USA) supplemented with 10% fetal calf serum (Gibco, New York, NY, USA) and 1X Antibiotic-Antimycotic (Gibco, New York, NY, USA). Cells were counted in a Neubauer chamber and assessed for viability with trypan blue. To obtain the antigen, subsequently, the cell pellet was resuspended in cold protein lysis buffer (1% Nonidet P-40 (Sigma, St. Louis, MO, USA), 150 mM NaCl (JT Baker, Phillipsburg, NJ, USA), 10 mM Tris-HCl (Sigma, St. Louis, MO, USA) pH 7.6, 10 mM PMSF (Sigma, St. Louis, MO, USA) 2 mM EDTA (Sigma, Steinheim, Germany), Protinin A (Sigma, St. Louis, MO, USA) 1 µg/mL, Pestatin A (Sigma, St. Louis, MO, USA), and 1 µg/mL y Leupeptin A (Sigma, St. Louis, MO, USA)) [64] and was broken by freezing for 30 min. The concentration of total antigen (Ag) was determined on a UV-Visible spectrophotometer at 280 nm Epoc (Biotec Instruments, Winooski, VT, USA).

### 4.3. Generation of Anti-T. cruzi Antibodies In Vivo with New Zealand Rabbits

Six-week-old rabbits were obtained from the Animal facility of the Children’s Hospital of Mexico Federico Gomez (Mexico City, Mexico). The animal study protocol was approved by the Ethics Committee of the Children’s Hospital of Mexico Federico Gomez (HIM-2014-034) and follows the guidelines for animal experiments of Mexico. Antibody induction was performed using proteins obtained from epimastigotes of CLB as the immunogen. At time 0, the rabbits were injected via intradermal with 2 mg of Ag dissolved in 150 µL physiological solutions, emulsified with 150 µL Freud’s complete adjuvant (Sigma, St. Louis, MO, USA); at day 15, they were injected via intradermal with 2 mg of Ag dissolved in 150 µL physiological solution, emulsified with 150 µL Freud’s complete adjuvant (Sigma, St. Louis, MO, USA). At day 30, the rabbits were injected intravenously with 0.25 mg Ag dissolved in 150 µL physiological solution; at day 31, they were injected intravenously with 0.50 mg Ag dissolved in 150 µL physiological solution; at day 32, they were injected intravenously with 0.50 mg Ag dissolved in 150 µL physiological solution. The total collection of the serum was on day 39. 

### 4.4. Purification of Anti-T. cruzi Antibodies by Affinity Chromatography with Protein A/G

IgG purification followed the manufacturer’s protocol from Thermo Scientific, using affinity chromatography with protein A/G using Spin Columns (Thermo Scientific, Rockford, IL, USA). The concentrations of purified antibodies were quantified by measuring the absorbance of each fraction at 280 nm in an Epoch (Biotec Instruments, Winooski, VT, USA) UV/Vis spectrophotometer.

### 4.5. Determination of Anti-T. cruzi Antibodies Recognition with SUPB15 by FC

For evaluating the recognition of anti-*T. cruzi* antibodies by FC analyses, the line cell SUPB15 was used. Free cell sites were blocked with 1 mg/100 µL/tube of IgG human (Jacson Inmunology Reserch, West Grove, PA, USA) for 15 min. Anti-*T. cruzi* antibodies were used at a dilution of 1:300 for 30 min. Reactivity was detected using specific anti-rabbit IgG antibodies conjugated to Alexa Fluor 488 (Invitrogen, OR, USA) diluted 1:300 for 30 min [65]. Cells were acquired using a BD Facs Canto ll (BD Biosciences, San Jose, CA, USA) flow cytometer, and the data were analyzed using FlowJo v10.0.7 (Tree star, Ashland, OR, USA) software. Mouse monoclonal Ab (clone: NCL/902, NeoBiotechnologies, cat. 4691-MSM1-P1) was used as a positive control for nucleolin.

### 4.6. CDC on SUP15 by Anti-T. cruzi Antibodies

The target cells were the SUPB15 cell line. The complement was obtained from healthy rabbits. CDC were evaluated by flow cytometry analyses by two independent stainings. First, we stained proliferating SUPB15 cells with Orange CMRA (Invitrogen, OR, USA) for 30 min. Subsequently, 500,000 SUPB15 cells were incubated for 4 h at 37 °C at 5% CO_2_ in the presence of 15% rabbit complement and a 1:300 dilution (8.8 µg/µL) of the anti-*T. cruzi* antibody. Finally, Blue LIVE/DEAD (Invitrogen, OR, USA) reagent staining was used to confirm cytotoxicity [66]. Cells were acquired using a (Becton Coulter, New York, NY, USA) FC, and the data were analyzed using FlowJo v10.0.7 (Tree star, Ashland, OR, USA) software. Mouse monoclonal Ab (clone: NCL/902, NeoBiotechnologies, cat. 4691-MSM1-P1) was used as a positive control for nucleolin.

### 4.7. Identification of SUP15 Proteins Recognized by Anti-T. cruzi Antibodies by WB

To identify the proteins of the SUPB15 that are recognized by anti-*T. cruzi* antibodies, WB and mass spectrometry were performed. The separating gel was prepared with 12% acrylamide-bisacrylamide (Sigma, Amsterdam, The Netherlands) and the concentrator gel with 4% acrylamide-bisacrylamide. Samples were prepared 1:5 with sample buffer, under reducing conditions. The voltage was set to 200 V (constant) for 50 min. The electrophoresis gel was placed on a 0.2 µM nitrocellulose membrane (Amersham Biosciences, Region, UK) and, in the Trans-Blot-Transfer (Bio-Rad, Hercules, CA, USA) chamber cassette, the voltage was conditioned at 100 V for 2 h. To verify the proper transfer of proteins to the nitrocellulose membrane, the membrane was stained with 0.2% Ponceau red and washed with water until the dye was completely removed. The dilution used for the anti-*T. cruzi* antibodies was 1:100 overnight. The rabbit anti-IgG antibody coupled to horseradish peroxidase (Sigma, Jerusalem, Israel) was 1:1000 diluted for 2 h. The blot was developed with 4-CN (Sigma, USA) and H_2_O_2_ (ProdQuimMonterrey, Monterrey, Mexico). Once the fragment of interest was identified in the Western blot, an electrophoresis gel was run and the fragment of interest was cut out.

### 4.8. Mass Spectrometry

Preparative gel electrophoresis was performed and the selected gel fragments were sent to the University Laboratory of Proteomics IBT-UNAM (Morelos, Mexico) for analysis in a quadrupole-TOF Impact II (Buker Daltonics, Billerica, MA, USA) mass spectrometer [67]. Mass spectra were processed using Data Analysis software 5.0 (Bruker Daltonics, Billerica, MA, USA) and compared against the Swiss Prot protein database using the Mascot search engine.

### 4.9. Identification of Nucleolin in SUPB15 by FC

To identify nucleolin in SUPB15 by FC analysis, free cell sites were blocked with 1 mg/100 µL/tube of IgG human (Jacson Inmunology Reserch, West Grove, PA, USA) for 15 min. The anti-*T. cruzi* antibodies were used 1:300 for 30 min; after washing, we added anti-rabbit IgG antibodies conjugated to Alexa-Fluor 647 (Invitrogen, OR, USA) diluted 1:300 for 30 min [65]. Cells were acquired using a BD Facs Canto ll (BD Biosciences, San Jose, CA, USA) FC, and the data were analyzed using FlowJo v10.0.7 (Tree star, Ashland, OR, USA) software.

## Data Availability

Data are contained within the article.
